# Usefulness of Urinary Creatinine/Urea Nitrogen Ratio as Indicator of Body Protein Catabolism in Dogs Fed Low Protein Diets

**DOI:** 10.3389/fvets.2019.00449

**Published:** 2019-12-10

**Authors:** Shushi Yamamoto, Yoshiyuki Ohta, Etsuko Hasegawa, Shiori Hashida, Yasuyuki Kaneko, Shinya Mizutani, Benedict Huai Ern Ong, Kiyokazu Naganobu, Shidow Torisu

**Affiliations:** ^1^Faculty of Agriculture, Veterinary Teaching Hospital, University of Miyazaki, Miyazaki, Japan; ^2^Laboratory of Applied Biochemistry, Department of Animal Science, Faculty of Applied Life Science, Nippon Veterinary and Life Science University, Musashino, Japan; ^3^NST Inc., Saitama, Japan

**Keywords:** canine, urinary creatinine, urinary urea nitrogen, low protein diet, protein catabolism

## Abstract

Low protein diets (LPs) constitute a reportedly effective form of nutritional therapy for canine chronic kidney disease and cirrhosis. These diets have long been feared to result in reduced muscle mass due to protein catabolism. This adverse effect, however, remains largely unrecognized in veterinary medicine as there are no easily applicable catabolism indicators. Therefore, we focused on urinary creatinine, a metabolite of protein in the urine, and examined whether its ratio to urinary urea nitrogen (UCrn/UN) can be used to assess protein catabolism. In Experiment 1, we first consecutively fed seven healthy beagles an LP, standard protein (SP), and high protein (HP) diet for 1 week each and then measured the UCrn/UN ratio at 2-h intervals from fasting to 16 h post-prandially. We consequently found that the UCrn/UN ratio was significantly elevated in the LP pre-prandially and at all post-prandial measurement points (*P* < 0.01). No significant differences were observed between the SP and HP. Analysis of fasting plasma amino-acid concentrations revealed that the concentration of methionine was significantly lower in the LP than in the other diets (*P* < 0.05). Although the effects of this change in amino-acid concentration were unclear, the UCrn/UN ratio was considered having increased due to a deficiency in protein and/or amino acids during LP feeding. In Experiment 2, we continuously fed five healthy beagles an LP for 18 weeks and then measured the UCrn/UN ratio as described above. We also measured changes in body composition with computed tomography. At weeks 10 and 18, the fasting UCrn/UN ratio was significantly higher than it was prior to the start of the LP; however, post-prandially, the UCrn/UN ratio decreased to the point that the significant difference disappeared. Muscle mass decreased at weeks 10 and 18. These results suggest that the fasting UCrn/UN ratio could be used as an indicator of protein catabolism in LP feeding. Our experiments thus indicate that examination of potential increases in the UCrn/UN ratio 1 week after introduction of LP feeding to healthy dogs could enable detection of body protein catabolism in long-term feeding of LP before muscle breakdown occurs.

## Introduction

Dietary protein is an important nutrient for organisms. It is broken down into peptides and amino acids, which are then absorbed into the body. Through this process, proteins serve as an energy source, as the building blocks of the body, and a wide variety of other functions including regulating homeostasis as enzymes and hormones, transporting substances in the blood, and enabling gene expression. However, in certain diseases such as cirrhosis and chronic kidney disease (CKD), a nitrogen load occurs even with the typical required protein intake, thereby sometimes exacerbating the disease state. Therefore, when cirrhosis or CKD develops, diet therapy typically restricts protein to prevent hepatic encephalopathy, alleviate uremia, inhibit the progression of renal impairment, and correct metabolic acidosis.

Many previous studies, in both human and veterinary medicine, have reported on the efficacy of low protein diets (LPs) as a diet therapy (1–3). However, the efficacy of LPs is sometimes disputed. Investigation is ongoing regarding clear protein restriction criteria and the optimal timing for starting protein restriction (4–6). The large-scale human Modification of Diet in Renal Disease Study reported results that cast doubt on the efficacy of LPs for treating CKD (7). In addition, recent evidence has emphasized the risk of protein-energy malnutrition (protein-energy wasting) in LPs (8–10). In veterinary medicine as well, long-term feeding of an LP has been claimed to potentially result in protein catabolism, particularly in the form of reduced muscle mass (6). However, this assertion has not been sufficiently verified, and the harmful effects of LP therapy have not been made evident. One reason given for this absence of verification is that body protein catabolism is difficult to determine. Generally, reduced muscle mass and lean body mass (LBM) are known to be involved in the prognosis of various diseases (11, 12). Therefore, body protein catabolism must be detected and corrected at an early stage.

Muscle mass, which is a form of body protein, can be easily assessed with the body condition score (BCS) or muscle condition score (MCS). However, these methods are subjective and not conducive to elucidating detailed changes. Bioelectrical impedance analysis and dual-energy X-ray absorptiometry are now used to measure body composition in dogs and cats (13). However, due to environmental and cost issues, these methods are only used in research facilities. In addition, dual-energy X-ray absorptiometry lacks precision when used in dogs (14).

Urinary 3-methylhistidine (3-MH) is typically used in humans as a simpler method for assessing muscle protein breakdown (15). However, because urinary 3-MH is assessed with 24-h urine and fluctuates from day to day, urine must be collected for several days. Therefore, the inability to assure the integrity of measurement in dogs and cats constitutes a clinical obstacle. Additionally, unlike humans and cats, dogs excrete large amounts of 3-MH in feces, signifying that urinary 3-MH may not accurately reflect muscle breakdown in dogs (16).

We, therefore, focused on urea nitrogen (UN) and creatinine (Crn), which are also metabolites of protein (amino acids), as is 3-MH, but are easier to measure. After being metabolized in the body, nearly all amino acids that makeup proteins are ultimately converted to UN in the urea cycle and excreted in urine. Therefore, the production and excretion of UN fluctuates depending on the levels of protein intake and the degree of hyper-catabolism (17, 18). Thus, urinary UN (UUN) reflects the results of the metabolic kinetics of proteins derived from diet and body tissue (particularly muscle).

In contrast, urinary excretion of Crn is reportedly constant and unaffected by diet (19) because Crn is produced daily by non-enzymatic reactions from 1 to 2% of creatine (Cr) and/or creatine phosphate and is then excreted with almost no reabsorption or secretion by the renal tubules. Furthermore, as reported by Borsook et al., Cr synthesis is stable and mostly unaffected by protein intake (20). Therefore, urinary creatinine (UCrn) reportedly remains unaffected by exogenous factors and has been used to correct concentrations of urine constituents and estimate the glomerular filtration rate. However, Cr, creatine phosphate, and Crn are largely pooled (more than 90%) in skeletal muscle, which is the main component of body protein. Thus, the amount of Crn released and excreted in urine may increase when protein is catabolized. In humans, elevated UCrn has been observed in patients with severe conditions in a state of body protein catabolism such as after surgery or in chronic obstructive pulmonary disease (21, 22). In these studies, the levels of urinary 3-MH and UCrn decreased when the nutritional status improved with total parenteral nutrition. Other studies have reported that UCrn increases when dietary protein requirements are met. Crim et al. found that UCrn levels increased as a result of additional administration of arginine and glycine, which are amino acids involved in Cr synthesis (23). Therefore, we believe that UCrn, in similar fashion to UUN, fluctuates due to the effects of dietary protein (amino acids) and body protein catabolism.

Based on these notions, in the present study, we aimed to use these two urinary metabolic excretion products to establish a method for assessing body protein catabolism during the feeding of an LP in dogs. First, we examined whether feeding diets with different levels of protein to healthy dogs would cause UUN and UCrn to change in accordance with the level of protein intake. Next, we examined whether long-term feeding of an LP would lead to changes in UUN and UCrn and whether these are useful indicators of body protein catabolism. We also simultaneously examined whether feeding a commercially-available LP would actually result in catabolism and other negative effects, as well as changes in plasma amino-acid concentrations and body composition.

## Materials and Methods

### Animals

The animals used in this study were healthy beagles raised at the Veterinary Teaching Hospital, Faculty of Agriculture, University of Miyazaki. Prior to the experiments, all animals underwent general physical and blood tests, radiography, and ultrasonography, which showed no abnormalities. Animal management and the experiments were conducted in strict adherence to the University of Miyazaki's Regulations for the Management of Laboratory Animals. The present study was conducted with the approval of the University of Miyazaki Animal Care and Use Committee (approval number: 2018-013-03).

### Diets

The LP in the present study consisted of a commercially-available dietetic food designed for dogs with CKD (Renal Support, ROYAL CANIN JAPON, Inc., Tokyo, Japan). To enable comparisons, we also fed the dogs a standard protein diet (SP) and a high protein diet (HP). The SP was a commercially-available maintenance food for adult dogs (NAFCO Corp., Fukuoka, Japan) that meets the nutritional standards of the Association of American Feed Control Officials (AAFCO) (24). The HP was a dietetic weight management food (Satiety Support Special, ROYAL CANIN JAPON, Inc.). We calculated each dog's daily energy requirement (resting energy requirement [70 × body weight (BW)^0.75^ × 1.6] to determine the total feeding amount, which we divided into two feedings. The composition of each diet is shown in Table 1. To analyze amino-acid compositions, we hydrolyzed diets with 6 N HCl, neutralized them with 5 N NaOH, filtered them, and then diluted them with a pH 2.2 lithium citrate buffer.

**Table 1:** Nutrient and amino-acid composition of each diet.

**Nutrient (g/1,000 kcal)**	**Diet**^**a**^			**AAFCO maintenance minimum^**b**^**
	**LP**	**SP**	**HP**	
Protein	35.0	65.0	112.0	45.0
Fat	45.0	23.0	36.0	13.8
Carbohydrate	131.0	148.0	108.0	N/A
**Amino acid**				
Arginine	1.12	4.76	6.55	1.28
Histidine	0.61	1.71	1.96	0.48
Isoleucine	1.19	2.90	4.44	0.95
Leucine	3.98	6.94	9.99	1.70
Lysine	1.61	3.69	5.84	1.58
Methionine	1.33	1.34	2.21	0.83
Methionine-Cysteine	1.68	1.97	3.17	1.63
Phenylalanine-Tyrosine	2.76	5.87	10.52	1.85
Threonine	1.02	2.86	4.20	1.20
Valine	1.50	3.66	5.53	1.23
Proline	2.43	5.74	9.53	N/A
Glycine	1.21	5.46	8.22	N/A
Alanine	2.17	5.27	7.11	N/A
Serine	1.41	3.77	6.21	N/A
Aspartic acid^c^	2.02	6.53	7.96	N/A
Glutamic acid^d^	8.66	18.80	32.22	N/A
Energy (kcal/100 g)	399	310	267	N/A

a *LP, low protein diet; SP, standard protein diet; HP, high protein diet*.

b *Cited from Association of American Feed Control Officials (AAFCO) (24)*.

c *Includes asparagine*.

d *Includes glutamine*.

### Experimental Design

#### Experiment 1

This experiment was conducted with two male and five female beagles ranging in age from 3 years 5 months to 5 years 10 months with BWs of 10.0–14.2 kg. Each dog was fed an LP, SP, or HP for 1 week each and was then subjected to the experiment. In the experiment, after the dogs had fasted for 16 h, we collected urine after placing a 6 or 7 Fr silicone balloon catheter. We then removed the urine from the catheter and used ultrasonography to confirm that the dogs' bladders were empty. The catheter was retained in place until the end of the experiment. The above process of urine collection and removal was repeated every 2 h from fasting until 16 h post-prandially. Fasting blood samples were collected from either the jugular or the cephalic vein. Due to concerns that vigorous exercise would affect the measurements, the dogs rested on the day of the experiment.

#### Experiment 2

This experiment was conducted with two male and three female beagles ranging in age from 3 years 9 months to 4 years with BWs of 10.6–13.9 kg. Prior to the experiment, the dogs were fed an SP for 2 weeks. Afterward, we fed the dogs an LP for 18 weeks to examine the effects of long-term LP feeding. During this period, we measured the BW, BCS, and MCS. At 0, 4, 10, and 18 weeks, we collected urine from fasting to 16 h post-prandially and fasting blood samples using the same methods as in Experiment 1. We also conducted computed tomography (CT) scanning at 0, 4, 10, and 18 weeks to assess changes in body composition.

### Analysis

#### Urinalysis

Fasting urine samples were subjected to urinometry and physical property analysis with a urine test strip. Urine specific gravity was measured with PAL-DOG&CAT (ATAGO CO., LTD, Tokyo, Japan). The physical properties of urine were analyzed by directly dropping urine onto a urine test strip 10 UB (ARKRAY Inc., Kyoto, Japan) and then running a test with a thinka RT-4010 (ARKRAY Inc.). We measured glucose, protein, bilirubin, occult blood, pH, ketone bodies, nitrite, leukocytes, Crn, albumin, and the urine protein to the Crn ratio.

#### Complete Blood Count and Chemistry

A portion of each fasting blood sample was immediately placed into blood collection tubes containing EDTA-2K and subjected to a complete blood count with a pocH-100 iV (Sysmex Corporation, Hyogo, Japan). The remaining blood was placed into a heparin tube (FUJIFILM Corporation, Tokyo, Japan) and centrifuged at room temperature at 7,700 rpm for 6 min. Using a DRI-CHEM 7000 V (FUJIFILM Corporation), we measured the following in the plasma obtained from centrifuging: blood urea nitrogen (BUN), Crn, total protein, albumin, total cholesterol, triglycerides, and blood glucose.

#### Urine Creatinine and Urine Urea Nitrogen

Collected urine was cryopreserved at −80°C until measurement. After the urine was thawed at room temperature, UCrn was analyzed using a Creatinine Assay Kit (Cayman Chemical Company, Ann Arbor, MI, USA) utilizing the Jaffe reaction. UUN was measured using the urease-indophenol method utilizing the Berthelot reaction. The above measurements were then used to calculate the ratio of UCrn to UUN (UCrn/UN).

#### Serum Insulin and Leptin

At all measurement points in Experiment 2 (0, 4, 10, and 18 weeks), fasting blood samples were placed in serum-separating blood collection tubes and centrifuged at room temperature at 3,000 rpm for 15 min to yield serum. We then measured insulin in this serum with a chemiluminescent enzyme immunoassay at a commercial laboratory (SRL, Inc., Tokyo, Japan). Leptin was measured in duplicate using a Canine Leptin ELISA Kit (Millipore, Billerica, MA) according to its protocol.

#### Plasma Amino Acids

Blood for fasting amino-acid analysis was placed into blood collection tubes containing EDTA-2Na and centrifuged at 4°C at 3,000 rpm for 15 min. The resulting plasma was then cryopreserved at −80°C until measurement. Plasma amino-acid concentrations were analyzed as recommended by Fujimura et al. (25) using an amino-acid analyzer (Shimadzu LC-20A, Shimadzu Corporation, Kyoto, Japan).

### CT Scanning and Image Processing

After fasting for 16 h, the dogs were sedated with an intravenous mixture of midazolam 0.2 mg/kg and butorphanol 0.2 mg/kg. Next, after intravenous administration of propofol 0.3 mg/kg, an endotracheal tube was inserted, and anesthesia was maintained via inhalation with isoflurane and 100% O_2_. For CT scanning, we acquired 1 mm-thick images at 120 kV and 200 mA using a 16-row multislice CT machine (Aquilion, Toshiba Medical Systems, Otawara, Japan). Images were analyzed using AZE VirtualPlace Lexus 64 (AZE Ltd, Tokyo, Japan). Components abstracted in the fat condition were removed from the 3D-reconstructed whole-body images, and the volume of the remaining portion was calculated as the LBM. Areas of fat were selected with the CT attenuation value set at −190/−30 Hounsfield units (HU) (26). To eliminate errors in the analysis, we subtracted air (intestinal gas) and urine by extracting air at an attenuation value of −1,000/−500 HU and performing contour extraction of urine from the transverse section. Moreover, based on a previous study (27), we measured the muscle and fat areas in the respective HU conditions (0/100 HU, −190/−30 HU) in sections of thoracic vertebrae 6, 9, and 12 (T6, 9, 12) and lumbar vertebrae 3 and 5 (L3, 5), where the spine can be observed most prominently, and of the femur at the level of the ischial arch. The muscle mass area was calculated by subtracting the splanchnic region after extraction at an attenuation value of 0/100 HU.

### Statistical Analysis

All data were analyzed with PROC MIXED including in the model the effects of diet and feeding duration as fixed effects for repeated measures using SAS University Edition (SAS Institute Inc., Cary, NC). For measurement comparisons, we used the least mean square algorithm and performed Tukey–Kramer multiple-comparison adjustment. We also used linear regression to analyze the correlation between UCrn and UUN in each diet in Experiment 1. Regression line slopes were compared with the analysis of covariance. For all analyses, the level of statistical significance was set at 5% (*P* < 0.05). Values were presented as mean ± standard error.

## Results

### Experiment 1

Table 1 lists the compositions of the diets used in Experiment 1. We did not observe any significant changes in BW in the 1 week of feeding an LP, SP, or HP (LP, 11.7 ± 0.6 kg; SP, 11.6 ± 0.5 kg; HP, 11.4 ± 0.6 kg). We also did not observe any differences in the BCS or MCS or changes from before to after eating. The urine specific gravity and urinalysis results were unremarkable. We did not observe abnormalities or differences associated with diet in the fasting complete blood count. In the blood chemistry analysis, BUN in the LP, SP, and HP conditions was 7.5 ± 0.6, 12.1 ± 0.9, and 14.7 ± 0.6, respectively (reference interval, 9.2–29.2 mg/dl). Thus, BUN was significantly lower in the LP condition (*P* < 0.01). However, no differences were observed in any other items.

**Figure 1:**
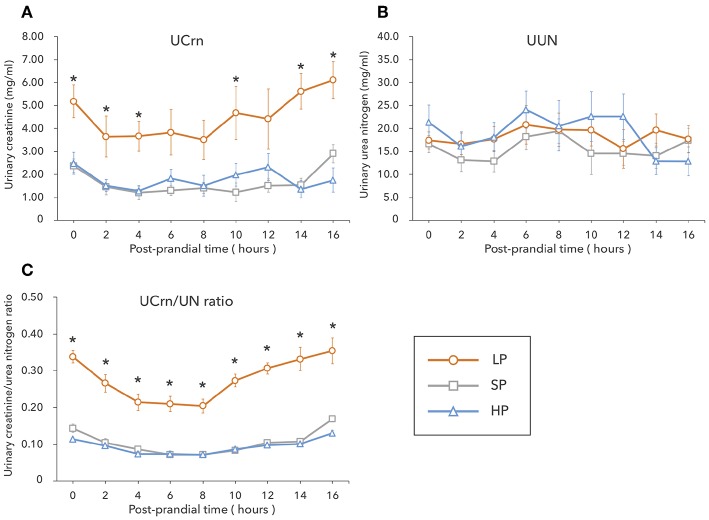
In Experiment 1, dogs were fed a low-protein diet (LP), a standard protein diet (SP), and a high-protein diet (HP) for 1 week each, after which the levels of creatinine **(A)** and urea nitrogen **(B)** excreted in urine were measured from fasting to 16 h post-prandially. The urinary creatine/urea nitrogen ratio **(C)** was calculated from the results of **(A,B)**. Values are listed as mean ± standard error for seven dogs. ^*^Indicates a significant difference with SP (*P* < 0.05).

In the LP condition, urinary Crn was overall high from fasting to 16 h post-prandially, showing significant differences with the SP and HP conditions at several timepoints. No significant differences were observed in UCrn between the SP and HP conditions. In contrast, UUN did not differ among diets (Figures 1A,B). The correlation between UCrn and UUN was significant in all diets (LP: *y* = 0.27*x* + 0.06, *R*^2^ = 0.79, *P* < 0.01; SP: *y* = 0.10*x* + 0.09, *R*^2^ = 0.59, *P* < 0.01; HP: *y* = 0.09*x* + 0.01, *R*^2^ = 0.79, *P* < 0.01). When we compared the regression line slopes, we did not observe differences between the SP and HP conditions (*P* = 0.16), but we did find a significant difference between the LP and SP conditions, as well as between the LP and HP conditions (*P* < 0.01). The UCrn/UN ratio was significantly higher in the LP than in the SP and HP conditions at all measurement points from fasting to 16 h post-prandially (*P* < 0.01) (Figure 1C). No significant differences were observed in the UCrn/UN ratio between the SP and HP conditions at any measurement point.

Next, we measured fasting plasma concentrations of the essential and non-essential amino acids that comprise proteins in the body, as well as the levels of 3-MH. We then compared these concentrations among diets (Table 2).

**Table 2:** Fasting plasma amino acid concentration after feeding with each diet for 1 week (Experiment 1).

**Amino acid^**1**^ (nmol/ml)**	**Diet**		
	**LP**	**SP**	**HP**
Valine	179.4 ± 18.5^ab^	163.7 ± 11.2^a^	216.7 ± 30.2^b^
Leucine	167.2 ± 45.8	180.0 ± 16.3	166.0 ± 57.3
Isoleucine	69.3 ± 8.7	60.5 ± 4.6	73.3 ± 9.1
Phenylalanine	67.6 ± 6.9	65.0 ± 4.1	80.1 ± 4.1
Tyrosine	49.4 ± 4.6^a^	47.3 ± 3.1^a^	63.5 ± 2.6^b^
Methionine	36.7 ± 8.0^a^	57.9 ± 3.7^b^	72.5 ± 4.6^b^
Cysteine^2^	3.2 ± 0.8	3.8 ± 0.5	4.1 ± 0.6
Threonine	170.7 ± 12.7^a^	162.8 ± 15.0^a^	289.1 ± 43.0^b^
Serine	143.7 ± 18.5	135.4 ± 15.0	172.4 ± 20.2
Glycine	214.0 ± 19.3^a^	210.6 ± 11.2^a^	304.8 ± 29.0^b^
Aspartic acid^3^	5.8 ± 0.6^a^	3.6 ± 0.5^b^	4.3 ± 0.3^b^
Glutamic acid^4^	189.5 ± 58.9	178.3 ± 57.3	194.3 ± 58.5
Histidine	74.2 ± 19.7	89.5 ± 4.7	115.9 ± 12.0
Arginine	100.6 ± 19.0^a^	107.8 ± 16.7^a^	175.0 ± 26.9^b^
Lysine	195.9 ± 27.7^a^	154.4 ± 25.9^b^	214.6 ± 27.7^a^
Proline	183.4 ± 30.4	195.4 ± 18.3	190.5 ± 18.8
Alanine	239.1 ± 55.9	238.0 ± 61.8	217.9 ± 63.4
Taurine	146.1 ± 12.9^a^	84.3 ± 6.1^b^	99.3 ± 23.7^b^
3-methylhistidine	18.5 ± 9.7	9.9 ± 1.2	11.3 ± 2.6

*Values are mean ± standard error*.

a, b *Different superscript letters in the same row indicate a significant difference (P < 0.05)*.

1 *Tryptophan is not included in the results due to an error in measurement*.

2 *Includes cysteine*.

3 *Includes asparagine*.

4 *Includes glutamine*.

Methionine was the only amino acid with a significantly lower concentration in the LP than in the SP and HP conditions. In contrast, concentrations of aspartic acid and taurine were higher in the LP than in the SP and HP conditions.

### Experiment 2

BW increased significantly at 10 weeks but returned to its original level at 18 weeks. In contrast, the BCS was slightly elevated at 18 weeks (Table 3) and MCS remained unchanged. We observed no changes over time in the urinalysis results. Regarding blood chemistry, total cholesterol increased (*P* < 0.01), but the levels remained within the normal range (0 weeks, 153.1 ± 8.1; 4 weeks, 251.4 ± 7.9; 10 weeks, 256.4 ± 18.1; 18 weeks, 220.2 ± 14.6 mg/dl; reference interval, 111–312 mg/dl). As in Experiment 1, BUN significantly decreased due to the feeding of an LP (*P* < 0.01). However, no significant differences were observed among the feeding durations (0 weeks, 12.1 ± 0.9; 4 weeks, 8.3 ± 0.3; 10 weeks, 7.2 ± 0.2; 18 weeks, 6.2 ± 0.5 mg/dl).

**Table 3:** Changes in body composition and serum hormone during long-term feeding of a low-protein therapeutic diet (Experiment 2).

	**Feeding duration**			
	**0 weeks**	**4 weeks**	**10 weeks**	**18 weeks**
Body weight (kg)	12.5 ± 0.5^a^	12.3 ± 0.4^a^	13.4 ± 0.4^b^	12.6 ± 0.5^a^
BCS (/9)	5.0 ± 0.0^a^	5.0 ± 0.0^a^	5.2 ± 0.1^a^	6.0 ± 0.1^b^
**CT analysis**				
Lean body mass (L)^1^	8.2 ± 0.5^a^	8.0 ± 0.5^ab^	7.7 ± 0.4^b^	7.9 ± 0.4^b^
Fat mass (L)	1.9 ± 0.4^a^	2.3 ± 0.4^b^	3.0 ± 0.4^c^	3.4 ± 0.4^d^
**Muscle area (cm**^**2**^**)**				
T6	55.6 ± 3.6^a^	52.7 ± 3.4^ab^	50.4 ± 3.8^b^	51.4 ± 3.9^b^
T9	42.3 ± 3.1^a^	38.6 ± 2.4^b^	37.2 ± 2.6^b^	37.4 ± 2.3^b^
T12	32.5 ± 2.3^a^	31.8 ± 1.7^a^	30.5 ± 1.8^a^	29.8 ± 1.9^b^
L3	38.5 ± 2.6	36.5 ± 1.8	35.5 ± 2.0	36.8 ± 1.8
L5	41.3 ± 2.6^a^	38.4 ± 2.2^b^	37.0 ± 1.9^b^	37.9 ± 1.9^b^
Femur	104.7 ± 5.3^a^	98.5 ± 2.9^a^	97.8 ± 2.9^b^	98.3 ± 3.7^a^
**Fat area (cm**^**2**^**)**				
T6	27.4 ± 6.3^a^	34.2 ± 6.8^b^	44.4 ± 7.1^c^	50.5 ± 7.4^d^
T9	27.3 ± 6.5^a^	32.8 ± 6.6^b^	42.9 ± 6.9^c^	49.6 ± 7.5^d^
T12	25.7 ± 5.4^a^	32.5 ± 5.6^a^	45.8 ± 6.0^b^	50.0 ± 5.5^b^
L3	38.2 ± 10.3^a^	51.3 ± 10.8^b^	60.7 ± 9.7^c^	72.5 ± 11.0^d^
L5	36.5 ± 8.9^a^	47.5 ± 8.7^b^	60.8 ± 8.5^c^	68.1 ± 9.4^d^
Femur	14.4 ± 3.1	14.7 ± 3.4	19.2 ± 3.9	23.9 ± 4.2
**Serum hormone**				
Insulin (μU/ml)	11.5 ± 1.5	13.0 ± 2.8	18.4 ± 5.4	10.2 ± 1.3
Leptin (ng/ml)	1.2 ± 0.4^a^	2.7 ± 0.9^a^	3.9 ± 1.3^b^	2.7 ± 0.8^a^

*Values are mean ± SE*.

a, b, c, d *Different superscript letters in the same row indicate a significant difference (P < 0.05)*.

1 *Whole body minus body fat, urine, and intestinal gas*.

Table 3 shows changes in body composition associated with long-term LP feeding. LBM decreased by ~6% over 10 weeks and subsequently reached equilibrium at 18 weeks. In contrast, fat mass continuously significantly increased until 18 weeks. In all sections except for L3, the muscle area decreased by 6.5–12% at 10 or 18 weeks. The fat area increased as feeding duration progressed in all except for the femoral section. Serum insulin levels showed no statistically significant changes after LP feeding began. Serum leptin levels increased following the start of the LP and were significantly elevated at 10 weeks.

**Figure 2:**
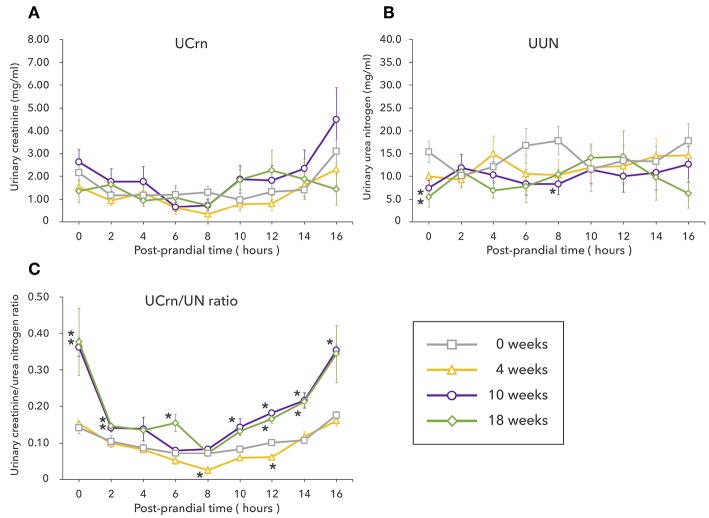
Experiment 2 involved long-term feeding of a low-protein diet. The graphs show changes in the levels of creatinine **(A)** and urea nitrogen **(B)** excreted in urine as well as changes in the urinary creatine/urea nitrogen ratio **(C)** from fasting to 16 h post-prandially at different feeding durations. Values are listed as mean ± standard error for five dogs. ^*^Indicates a significant difference with 0 weeks (*P* < 0.05).

Unlike in Experiment 1, UCrn showed no significant elevation pre-prandially or post-prandially after LP feeding begun. Pre-prandial UUN at 10 and 18 weeks was significantly lower than that at 0 weeks and remained low post-prandially after LP feeding began. However, no significant differences were observed between feeding durations (Figures 2A,B). The results for the UCrn/UN ratio differed based on feeding duration (Figure 2C). Unlike in Experiment 1, the UCrn/UN ratio at 4 weeks was nearly identical to that at 0 weeks. At 10 and 18 weeks, the UCrn/UN ratio was significantly elevated pre-prandially, decreased rapidly post-prandially, and, at 8 h post-prandially, was nearly identical to that at 0 weeks. All feeding durations showed the same post-prandial pattern of changes; the UCrn/UN ratio decreased until 8 h post-prandially and then increased again.

Plasma concentrations of 3-MH released during protein breakdown showed no changes from 0 to 18 weeks. The concentrations of most other amino acids increased after 0 weeks but showed no significant differences (Table 4).

**Table 4:** Fasting plasma amino acid concentration during long-term feeding of a low-protein therapeutic diet (Experiment 2).

**Amino acid^**1**^(nmol/ml)**	**Feeding duration**			
	**0 weeks**	**4 weeks**	**10 weeks**	**18 weeks**
Valine	157.2 ± 13.1	246.8 ± 54.0	169.5 ± 32.6	171.9 ± 35.6
Leucine	160.2 ± 13.6	219.9 ± 51.0	204.6 ± 37.1	168.5 ± 38.2
Isoleucine	60.9 ± 6.6	89.5 ± 18.6	68.0 ± 12.2	69.9 ± 15.4
Phenylalanine	68.9 ± 3.8	108.9 ± 20.0	90.1 ± 17.3	77.2 ± 13.3
Tyrosine	49.2 ± 3.1	73.5 ± 11.7	70.2 ± 11.6	56.9 ± 9.4
Methionine	62.1 ± 2.7	86.9 ± 18.6	105.7 ± 19.7	76.4 ± 19.2
Cysteine^2^	4.0 ± 0.7	5.1 ± 0.8	6.8 ± 0.2	6.8 ± 1.3
Threonine	154.2 ± 19.0	270.5 ± 86.6	192.0 ± 32.9	177.3 ± 35.7
Serine	139.2 ± 17.2	198.4 ± 53.0	165.9 ± 21.2	181.1 ± 47.7
Glycine	210.7 ± 12.6	387.3 ± 96.0	270.2 ± 36.5	346.1 ± 84.1
Aspartic acid^3^	3.6 ± 0.5	5.8 ± 1.3	3.5 ± 0.3	4.5 ± 1.1
Glutamic acid^4^	225.3 ± 69.9	360.3 ± 142.3	148.4 ± 48.8	200.0 ± 77.7
Histidine	90.5 ± 4.9	139.2 ± 24.4	107.7 ± 17.3	93.4 ± 16.3
Arginine	86.4 ± 6.6	150.7 ± 26.0	122.8 ± 22.1	128.1 ± 24.7
Lysine	154.4 ± 25.9	288.4 ± 108.7	174.5 ± 37.8	186.5 ± 44.9
Proline	207.9 ± 18.9	311.3 ± 69.1	326.2 ± 42.4	320.2 ± 82.7
Alanine	238.0 ± 61.8	576.7 ± 227.7	332.8 ± 56.0	449.3 ± 143.0
Taurine	84.7 ± 8.3^a^	182.2 ± 32.4^b^	115.5 ± 14.2^ab^	141.0 ± 28.4^ab^
3-methylhistidine	9.1 ± 1.5	10.0 ± 2.6	8.1 ± 2.3	8.8 ± 2.4

*Values are mean ± SE*.

a, b *Different superscript letters in the same row indicate a significant difference (P < 0.05)*.

1 *Tryptophan is not included in the results due to an error in measurement*.

2 *Includes cysteine*.

3 *Includes asparagine*.

4 *Includes glutamine*.

## Discussion

Proteins, lipids, and carbohydrates are all needed by organisms but proteins fundamentally differ from lipids and carbohydrates. Lipids can be synthesized into glucose via glycerol and dihydroxyacetone phosphate, while carbohydrates can be synthesized into cholesterol via pyruvate by glycolysis. Thus, lipids and carbohydrates can be re-synthesized from one another after they are broken down in the body. In contrast, the only nutrients that can function as proteins are proteins. In addition, excess lipids and carbohydrates can be easily stored as body fat, whereas excess protein is only stored in extremely small quantities. Therefore, proteins are irreplaceable and important dietary nutrients. Protein restrictions have thus been indicated to potentially cause problems such as loss of LBM, hypoalbuminemia, opportunistic infection, and disturbances in neural function (12, 28, 29). However, few studies have described adverse events such as the above in small-animal clinical practice (11) probably because of the difficulty in assessing protein nutritional status. Therefore, we examined whether UUN and UCrn can be used to assess protein and amino-acid deficiencies in a simple fashion.

In Experiment 1, we examined whether UUN and UCrn reflect the effects of different levels of dietary protein. The levels of UN production and excretion are considered to be positively correlated with levels of dietary protein (17). Therefore, UUN is sometimes used to estimate protein intake (30, 31). In the present study, BUN levels changed in accordance with protein levels. However, contrary to our prediction, UUN did not change based on diet. In a study by Peil et al. in which rats were fed an SP (24%) and an LP (8%), fractional excretion of urea decreased by half in the 8% LP compared to the 24% SP (32). Conversely, in the present study, the difference in protein levels among diets was small (~22 vs. 15%) compared with the study, which may explain the absence of an effect on UN excretion. Furthermore, recent evidence has suggested that as dietary protein levels change, attendant changes occur in lower urinary tract epithelial permeability and the reabsorption of the UN across the urinary tract epithelia (33). The above findings indicate that UN excretion may not correlate with the level of dietary protein.

In contrast to UUN, the UCrn concentration changed with dietary protein level. Ever since Folin proposed that Crn metabolism is unaffected by dietary protein (17), UCrn had been considered to be unaffected by exogenous factors (18, 34). In addition, because Crn synthesis depends on muscle mass, UCrn was considered to be constant. However, as Crn measurement has become more precise, some studies on human subjects have reported that UCrn levels fluctuate based on the levels of protein intake (35, 36). Ritchey et al. reported a positive correlation between UCrn levels and nitrogen intake ranging from 3.54 to 14.12 g/day (35). In Experiment 1, UCrn increased only in the LP in contrast to the results of previous studies. In addition, the absence of a difference between the SP and HP showed that this change in UCrn only occurred when protein requirements were not met. These results cannot be explained solely by the metabolic kinetics of dietary protein (exogenous). One conceivable explanation is the increased release of Crn primarily from muscle and increased excretion of Crn affected greatly by body protein (endogenous) metabolism, particularly catabolism. In a study that examined UCrn in rats using diets containing various levels of protein and amino acids, Crn excretion in urine was highest when nitrogen intake was lowest (37). In another study with cats fed a low protein prescription diet similar to the LP in the present study, UCrn increased over time (38). The results of the present study revealed that increases in UCrn may reflect protein catabolism.

Another important finding of the present study is that UCrn reflects body protein catabolism in response to an LP more keenly than does UUN. Bingham et al. found that urinary nitrogenous excretions consisted almost entirely of UN when protein intake was normal, whereas UCrn accounted for a larger portion of urinary nitrogenous excretions when protein intake was low (39). Thus, when protein requirements are not met, UCrn increases relative to UUN. The results of the present study showed the same relationship between UUN and UCrn. Based on this relationship, we compared the UCrn/UN ratio among diets and found that it was significantly higher in the LP than in the SP and HP conditions at every measurement point from fasting to 16 h post-prandially. In addition, the complete absence of a difference in the UCrn/UN ratio between the SP and HP conditions showed that the UCrn/UN ratio does not change in response to dietary protein levels but instead only increases when protein requirements are not met. This result suggests that a high UCrn/UN ratio reflects a deficiency in protein metabolism requirements unrelated to diet, regardless of when urine is collected. The result also suggests that if the UCrn/UN ratio is used as an indicator, body protein catabolism during feeding of an LP can be assessed with spot urine. Assessing UCrn in combination with UUN rather than assessing UCrn alone may correct the error associated with urine volume at urine collection and thus increase the accuracy of spot urine-based assessments. Because UCrn is largely unaffected by diet, it has been typically used to correct urine concentration and urine volume error in spot urine-based assessment of urinary excretions. However, in the present study, our focus on changes in UCrn associated with an LP led us to diverge from convention and divide UCrn by UUN. This may enable the use of the UCrn/UN ratio as an indicator of body protein catabolism that can be assessed with spot urine.

Protein hyper-catabolism is conceivably the main cause of an elevated UCrn/UN ratio during LP feeding. However, excess dietary arginine has previously been shown to increase Cr synthesis and Crn excretion (40, 41). Therefore, we examined dietary amino-acid compositions. Biosynthesis of Cr, the precursor of Crn, begins with the production of guanidinoacetate from arginine and glycine in the kidneys, with arginine:glycine amidinotransferase as a catalyst (34, 42). In the next stage, which occurs primarily in the liver and is catalyzed by guanidinoacetate methyltransferase, guanidinoacetate is methylated to yield Cr. This methylation requires S-adenosylmethionine, which is derived from methionine, as a methyl donor. Based on this pathway, arginine, glycine, and methionine are known to be involved in fluctuations in Cr and Crn. Glycine is a non-essential amino acid with multiple synthesis and metabolic pathways in the body. Therefore, the most important dietary amino acids are the essential amino acids arginine and methionine. Hasegawa et al. found that UCrn excretion increased in a dose-dependent manner based on dietary levels of arginine and methionine (43). Although we compared the composition of amino acids in the LP, in which UCrn was elevated, to the amino-acid compositions in the SP and HP, we did not find larger quantities of arginine or methionine. This result indicated that increased Cr synthesis and Crn excretion are not caused by excessive intake of arginine or methionine.

Next, we compared plasma amino-acid concentrations among diets to determine whether the amino-acid kinetics in each diet is associated with Cr/Crn synthesis or protein catabolism. We consequently found that of all essential amino acids, methionine was the only one with an evidently lower concentration in the LP than in the other diets' conditions. Due to maintenance of amino-acid homeostasis in the body, plasma amino-acid patterns are reportedly not greatly affected by dietary amino-acid composition or intake levels (44). Regardless, the reduced plasma concentration of methionine associated with LP suggests a relative deficiency in dietary methionine and increased methionine consumption in the body. When dietary amino acids alone do not satisfy amino-acid requirements, the deficiency is believed to be generally supplemented by endogenous amino acids supplied via the breakdown of body protein (45). Due to this phenomenon, in the occurrence of hyper-catabolism to supplement methionine, endogenous arginine is simultaneously also supplied. Therefore, we cannot exclude the possibility that excretion of Crn in urine increased in the present study due to arginine surplus as a consequence of an increased supply of endogenous arginine rather than dietary (exogenous) arginine. However, the LP used in the present study contained levels of essential amino acids that did not involve extreme restrictions compared to the AAFCO maintenance minimums (26). Therefore, we cannot determine based on the present study alone whether the levels of methionine were in fact insufficient. However, regardless of significantly high plasma concentrations of arginine and glycine in the HP compared to the SP condition, UCrn and UCrn/UN were unaffected. Thus, methionine kinetics may be highly relevant to these changes. In addition, the non-essential amino acids serine and glycine are involved in the metabolic pathway from methionine to cysteine. Therefore, important aspects of dietary amino acids may include essential and non-essential amino-acid concentrations and the ratio of essential to non-essential amino acids. In any case, LP feeding unambiguously increased the UCrn/UN ratio, suggesting that body protein was catabolized.

In Experiment 2, we examined whether long-term feeding of a low protein therapeutic diet would result in sustained hyper-catabolism. While the level of protein in the LP did not meet the AAFCO maintenance minimum (26), the level of protein did meet the allowance recommended by the National Research Council (25.0 g/1,000 kcal) (46). Thus, protein restriction was not extreme, and energy requirements were met. Therefore, it would not be possible to observe the negative effects of LP based on appearance (i.e., BW and BCS). However, CT-based body composition analysis revealed a significant decrease in muscle mass in terms of both volume and sectional area at 10 weeks. This result suggests that long-term LP feeding resulted in negative protein balance and muscle protein breakdown. Even when energy is sufficient, a deficiency or imbalance in proteins and/or amino acids has been empirically proven to suppress body protein synthesis and trigger body protein breakdown (47, 48). While the risks of an LP have been informally debated, few studies have proven these risks in dogs and cats. Hall et al. found that LBM decreased in cats with early CKD (IRIS stages 1 and 2) fed a low protein therapeutic diet for 6 months (38). Similarly, the present study showed that canine body protein was catabolized due to feeding of a typical low protein therapeutic diet.

Plasma amino-acid concentrations in Experiment 2 did not significantly differ over time after 4 weeks. This result suggests that an insufficient supply of amino acids from the diet is supplemented by endogenous amino acids supplied by the breakdown of muscle. Thus, body-protein catabolism may have continued. Plasma 3-MH concentration represents fluctuations in muscle protein breakdown in several hours and reportedly indicates acute changes in protein metabolism (49). However, in the present study, we did not observe any evident differences in plasma 3-MH concentration associated with dietary protein level or feeding duration. The level of muscle-protein breakdown in the present study may not have been sufficiently large to be released in the blood and increase the plasma 3-MH concentration. In dogs, unlike in humans or cats, 3-MH is decarboxylated to form 3-methylhistamine. Thus, canine muscle protein breakdown may not be reflected in plasma 3-MH concentration (16). Therefore, the use of plasma 3-MH concentration as an indicator of canine protein metabolism may require further consideration.

Based on the reduction in muscle mass observed on the CT scans, we predicted that both UCrn and the UCrn/UN ratio would increase over time. However, UCrn did not change significantly. One conceivable explanation of why UCrn did not increase as it did in Experiment 1 is that the long experiment period precluded the degree of muscle breakdown that occurred shortly after LP feeding began. However, although the differences were not significant, UUN decreased over time after LP feeding began, suggesting that nitrogen excretion overall decreased. Despite this decrease in UUN, UCrn did not decrease over time, suggesting that UCrn was excreted as a result of hyper-catabolism. In contrast, the fasting UCrn/UN ratio was higher at 10 and 18 weeks, when muscle mass had decreased, than at 0 weeks. These results reconfirm that the fasting UCrn/UN ratio can serve as an indicator of body-protein catabolism during feeding of an LP, including long-term feeding. However, the UCrn/UN ratio at 4 weeks was not higher than that at 0 weeks, indicating that body protein catabolism may have been suppressed by then. When protein is restricted, reduction of protein turnover is considered triggering an adaptation to protein restriction (50–52). In a study on canine protein catabolism in which healthy dogs were fed a 12% protein diet for 3 weeks, an examination of the kinetics of isotope-labeled leucine revealed decreases in both body protein synthesis and breakdown (53). While the LP used in the present study included 14% protein, a similar metabolic adaptation conceivably occurred at 4 weeks, thereby reducing protein requirements and leading to protein sufficiency despite the low level of dietary protein intake. Moreover, the UCrn/UN ratio at 8 h post-prandially was significantly lower at 4 weeks than at 0 weeks. This decrease may have been due to a relative deficiency of amino acids involved in Cr–Crn synthesis. However, this cannot be proven based on the present study alone. From 4 to 10 weeks, protein supply most likely did not meet the demand for protein needed for biological maintenance. Humbert et al. found that canine metabolic adaptation is possible with a nitrogen requirement for maintenance of 0.41–0.55 g N/kg BW^0.75^/day (51). Although the LP in the present study comfortably met this requirement (0.74 g N/kg BW^0.75^/day), the reduction in muscle mass and the results for the UCrn/UN ratio suggested that body protein was catabolized. While feeding in the study by Humbert et al. lasted for 2 weeks, Experiment 2 in the present study involved feeding for 18 weeks. Thus, the requirement cited by Humbert et al. is insufficient for long-term feeding of an LP. However, unlike in Experiment 1, the post-prandial UCrn/UN ratio at 10 and 18 weeks decreased to the same level observed at 0 weeks, suggesting that even the level of protein in an LP suppresses catabolism. Regardless, long-term feeding of an LP ultimately resulted in reduced muscle mass, indicating that the increase in the UCrn/UN ratio starting from 8 h post-prandially was due to hyper catabolism associated with dietary protein deficiency and that LP feeding cannot completely suppress protein catabolism throughout the post-prandial period.

Another effect of LP in the present study was an increase in body fat. Wakshlag et al. also found that dogs that consumed an LP lost LBM and gained body fat (54). To prevent protein from being used as a source of energy, protein-restricted diets take energy density into account and therefore contain increased levels of carbohydrates and lipids. Although fasting serum insulin levels were normal in the present study, due to the high amounts of carbohydrates in the LP (energy ratio ≥ 50%), the anabolic stimulus provided by increased insulin secretion may have led to fat accumulation. Obesity and basic studies with humans have also found that dietary protein levels affect energy expenditure (55, 56). In this study, LP feeding may have reduced protein turnover and consequently reduced ATP consumption. Previous reports indicate that ATP consumed for protein turnover accounts for ~20% of total body energy consumption (57). Thus, in Experiment 2, it is possible that the excess energy was stored as body fat.

Leptin, a peptide hormone produced by adipose tissue, suppresses appetite, increases energy expenditure via the hypothalamus, and regulates the storage of body fat (58). Therefore, plasma leptin concentration is considered to be positively correlated with body fat content (59). In the present study, plasma leptin concentration increased as body fat increased. Based on the above, protein restriction unmistakably led to increased body fat. Considering that obesity is associated with insulin resistance and cardiovascular disease in dogs and in humans (58), this increase in body fat must be recognized as a negative effect of LP therapy.

In light of these changes in body composition, caution is required when using an LP for early CKD and chronic liver disease (CLD) in dogs. In small-animal veterinary medicine, nutrition therapy is mainly based on commercially-available dietetic food because it is easy to use and stably supplied. However, commercially-available dietetic food must be used with caution, as it is not tailored to an individual organism's pathology and the associated convenience can lead to its careless use for long periods. Early protein restriction leads to loss of LBM, the progression of which is known in the medical field to lead to increases in disease prevalence and mortality (60, 61). In chronic diseases indicated for LP therapy, once muscle mass decreases, multiple problems related to age, dietary intake, and activity also occur. This concatenation of problems hinders the correction of reduced muscle mass and thus highlights the importance of developing the means to prevent muscle wasting associated with protein catabolism. When LP therapy begins, this muscle wasting can conceivably be prevented by assessing the amount of protein in the diet and its amino-acid composition to confirm that they would not trigger protein catabolism. Protein requirements in individual animals have been conventionally estimated using nitrogen balance. However, in LPs, muscle mass and body cell mass sometimes decrease even with a nitrogen equilibrium of zero (62). Thus, nitrogen balance is poorly suited to prevent body protein catabolism. The results of Experiments 1 and 2 in the present study suggest that the UCrn/UN ratio is a useful indicator of body protein catabolism during feeding of an LP. Thus, the UCrn/UN ratio can be used to qualitatively assess whether a diet would trigger hyper-catabolism in individual dogs and, potentially, to assess the risks posed by the LP. Regarding the timing of diet assessment, Rand et al. found that patterns of urinary nitrogen excretion had stabilized 4.5 days after subjects changed to an LP (63). In the present study, we were able to assess diets 1 week after they were changed. In addition, if the UCrn/UN ratio is assessed 1 week after a diet is changed, it can be assessed with spot urine both before and after eating. Therefore, the UCrn/UN ratio may also be highly useful as a point-of-care test. As for cases in which LP therapy is already underway, the results of Experiment 2 showed that the UCrn/UN ratio cannot be assessed in the first 4 weeks because protein metabolism adapts to the low protein intake. Instead, the UCrn/UN ratio should be assessed pre-prandially from 10 weeks onward. However, the point at which the UCrn/UN ratio can be assessed may differ depending on dietary protein levels and feeding conditions. This question cannot be conclusively answered based solely on the present findings. In any case, the most suitable means to prevent muscle breakdown in LP therapy may be to examine for an increased UCrn/UN ratio early in the diet, specifically at 1 week after the diet has begun.

The increase in UCrn in LP feeding is also interesting because, until now, UCrn has been considered to be stable and largely unaffected by diet. Therefore, UCrn was used to calculate Crn clearance, which reflects the glomerular filtration rate, and the urine protein/Crn ratio, which is an indicator of proteinuria. Polzin et al. showed that endogenous Crn clearance is not affected by dietary protein intake (64). However, the results of the present study suggested that those tests may lack precision due to changes in UCrn during feeding of an LP. In particular, renal function may be overestimated depending on LP feeding duration and when tests are performed. These issues may result in incorrect assessments of symptoms and therapeutic effects in dogs with CKD who are undergoing LP therapy. Going forward, assessments of symptoms and therapeutic effects must consider the effects of diet.

Limitations of the present study are that the duration of experiments were limited and that only healthy dogs were tested. Thus, the applicability of the UCrn/UN ratio is unclear in dogs with CKD and CLD undergoing LP therapy. CKD involves changes in glomerular filtration and reabsorption capacity, while CLD is highly likely to involve reduced Cr and UN. Therefore, clinical studies must be conducted on canine CKD and CLD cases. In addition, while the UCrn/UN ratio can be used to detect protein catabolism that occurs only during feeding of an LP, some cases of CKD and CLD already show protein hyper-catabolism and reduced muscle mass before LP therapy has begun. Therefore, thorough investigation must be conducted on the UCrn/UN ratio and other simple tests that can detect body protein catabolism in such cases. Furthermore, in some cases, progression of symptoms requires a low protein therapeutic diet despite the risk of body protein catabolism. A randomized clinical trial of low protein therapeutic diets for dogs with CKD showed such diets to be effective in terms of alleviating/delaying symptoms and prolonging survival (1). The amounts of phosphorus, sodium, and other nutrients are adjusted in these therapeutic diets. Thus, while the effects of low protein therapeutic diets likely do not derive from protein alone, they are an undeniably significant form of nutritional therapy. Wakshlag et al. indicated that downregulation of the ATP-dependent ubiquitin-proteasome pathway, a protein degradation pathway, may or may not occur depending on differences in the composition of essential amino acids in a protein diet, even in a 12% protein diet (54). Therefore, as protein catabolism can be suppressed by some dietary amino-acid compositions even during protein restriction, LPs must be designed with a focus on amino-acid composition.

In conclusion, our results demonstrate that the fasting UCrn/UN ratio can be used as an indicator of body protein catabolism associated with LP feeding. However, as protein metabolism may adapt depending on feeding duration, caution is required when using the UCrn/UN ratio as a dynamic indicator. When LP therapy is introduced, a comparison of the UCrn/UN ratio before and 1 week after the change in diet can be used to determine whether an individual dog's intake of dietary protein or amino acids meets their metabolic maintenance requirement and to assess protein catabolism before muscle mass decreases.

## Data Availability Statement

The datasets generated for this study are available on request to the corresponding author.

## Ethics Statement

The animal study was reviewed and approved by University of Miyazaki Animal Care and Use Committee (approval number: 2018-013-03).

## Author Contributions

SY, YO, and ST contributed to the design of the study and interpretation of results. SY performed the experiments and wrote the paper. YO, EH, and SH contributed to the lab analysis. YK, SM, BO, and KN contributed to the sample acquisition. ST revised the paper. All authors read and approved the final manuscript.

### Conflict of Interest

EH was employed by NST Inc. The remaining authors declare that the research was conducted in the absence of any commercial or financial relationships that could be construed as a potential conflict of interest.

## Abbreviations

3-MH: urinary 3-Methylhistidine
AAFCO: Association of American Feed Control Officials
BCS: body condition score
BUN: blood urea nitrogen
BW: body weight
CKD: chronic kidney disease
CLD: chronic liver disease
Cr: creatine
Crn: creatinine
CT: computed tomography
HP: high protein diet
LBM: lean body mass
LP: low protein diet
MCS: muscle condition score
SP: standard protein diet
UCrn: urinary creatinine
UCrn/UN: urinary creatinine/urea nitrogen
UN: urea nitrogen
UUN: urinary urea nitrogen.
